# Chondrogenic differentiation of human subchondral progenitor cells is affected by synovial fluid from donors with osteoarthritis or rheumatoid arthritis

**DOI:** 10.1186/1749-799X-7-10

**Published:** 2012-03-13

**Authors:** Jan Philipp Krüger, Michaela Endres, Katja Neumann, Bruno Stuhlmüller, Lars Morawietz, Thomas Häupl, Christian Kaps

**Affiliations:** 1 TransTissue Technologies GmbH, Charitéplatz 1, 10117 Berlin, Germany; 2 Tissue Engineering Laboratory, Department of Rheumatology and Clinical Immunology, Charité-Universitätsmedizin Berlin, Charitéplatz 1, 10117 Berlin, Germany; 3 Department of Pathology, Charité-Universitätsmedizin Berlin, Charitéplatz 1, 10117 Berlin, Germany; 4 Department of Rheumatology and Clinical Immunology, Charité-Universitätsmedizin Berlin, Charitéplatz 1, 10117 Berlin, Germany

**Keywords:** Cartilage regeneration, Chondrogenesis, Osteoarthritis, Synovial fluid, Microfracture, Rheumatoid arthritis, Stem cell

## Abstract

**Background:**

Microfracture is a first-line treatment option for cartilage repair. In microfracture, subchondral mesenchymal cortico-spongious progenitor cells (CSP) enter the defect and form cartilage repair tissue. The aim of our study was to investigate the effects of joint disease conditions on the *in vitro *chondrogenesis of human CSP.

**Methods:**

CSP were harvested from the subchondral bone marrow. CSP characterization was performed by analysis of cell surface antigen pattern and by assessing the chondrogenic, osteogenic and adipogenic differentiation potential, histologically. To assess the effect of synovial fluid (SF) on chondrogenesis of CSP, micro-masses were stimulated with SF from healthy (ND), osteoarthritis (OA) and rheumatoid arthritis donors (RA) without transforming growth factor beta 3.

**Results:**

CSP showed the typical cell surface antigen pattern known from mesenchymal stem cells and were capable of osteogenic, adipogenic and chondrogenic differentiation. In micro-masses stimulated with SF, histological staining as well as gene expression analysis of typical chondrogenic marker genes showed that SF from ND and OA induced the chondrogenic marker genes aggrecan, types II and IX collagen, cartilage oligomeric matrix protein (COMP) and link protein, compared to controls not treated with SF. In contrast, the supplementation with SF from RA donors decreased the expression of aggrecan, type II collagen, COMP and link protein, compared to CSP treated with SF from ND or OA.

**Conclusion:**

These results suggest that in RA, SF may impair cartilage repair by subchondral mesenchymal progenitor cells in microfracture, while in OA, SF may has no negative, but a delaying effect on the cartilage matrix formation.

## Background

Different cartilage regeneration strategies and techniques are used in clinical routine today. Especially, bone marrow stimulating techniques like pride drilling [1] and microfacture technique [2] are frequently used. Microfracture involved the debridement of damaged tissue down to the subchondral bone to induce bleeding, thus allowing mesenchymal progenitor cells derived from the subchondral bone, cortico-spongious progenitor cells (CSP) to enter the defect. These CSP are characterised by high proliferation capacity and the ability to differentiate into bone, cartilage and fat. Also CSP show the typical cell surface markers known from mesenchymal stem and progenitor cells, such as CD 73, CD 90, CD 105 and CD 166 [3-6]. The migration and recruitment of such CSP is mediated by cytokines and growth factors, also present in varying amounts in human synovial fluid (SF) [7-9]. These progenitor cells that reside in the subchondral bone form a non-hyaline cartilage repair tissue [10]. Additionally, there is evidence that the structure of the repair tissue formation may depend on the composition of SF. For example, SF from donors with trauma or osteoarthritis (OA) stimulated bovine chondrocytes to a higher extent of proteoglycan synthesis than the SF of rheumatoid arthritis (RA) donors [11]. In addition it has been shown that SF from acutely injured knees stimulated chondrogenesis, whereas SF from chronically injured knees inhibited chondrogenic differentiation [12]. It is also known that in both arthritic diseases (RA and OA) the SF contains inflammatory mediators such as cytokines, chemokines, matrix metalloproteinases (MMP), tumor necrosis factor-alpha (TNF-α), interleukins and growth factors which play a major role during the etiopathology of the disease. Also the protease and proteinase inhibitors TIMP1, TIMP2 and α_2_-macroglobulin (α_2_M) are involved in this process. But in RA patients the inflammatory mediators were increased compared to OA patients [13-19]. The protease and proteinase inhibitors were decreased in RA patients compared to OA patients [17,19]. However, in both diseases there is a clear correlation of an inflammatory mediator/proteinase inhibitor imbalance compared to healthy individuals, which have a balanced inflammation mediator/proteinase inhibitor ratio. It is also known that mesenchymal progenitor cells from patients with RA and OA have the similar chondrogenic potential as mesenchymal progenitor cells from healthy donors [20].

In summary, in both arthritic diseases (RA and OA) inflammatory mediators such as cytokines, chemokines, MMPs and growth factors play a major role during the onset and progression of the disease. In both diseases there is a clear agreement of an inflammatory mediator/proteinase inhibitor imbalance compared to healthy individual, which have an inflammatory mediator/proteinase inhibitor balance [17,19]. It is also known that mesenchymal progenitor cells from patients with RA and OA have the similar chondrogenic potential as mesenchymal progenitor cells from healthy donors (ND) [20]. Further, experiments showed that an OA environment does not impair cell migration compared to a healthy environment. In contrast, RA environment reduced the cell migration capacity of progenitor cells compared to OA and ND environment [8] and we have shown that inflammatory synovial fluid derived from donors with rheumatoid arthritis inhibits the chondrogenic differentiation sequence induced by the growth and differentiation factor TGFB3, transforming growth factor beta 3 [4]. To resemble more closely the clinical situation, the aim of the current study was to evaluate the effect of human synovial fluid from normal, rheumatoid arthritis and osteoarthritis donors on the chondrogenic differentiation of human subchondral cortico-spongious progenitor cells, without any external chondrogenic stimulus by recombinant growth factor TGFB3.

## Methods

### Isolation and cultivation of cortico-spongious progenitor cells (CSP)

Human CSP were isolated from subchondral cortico-spongious bone chips (n = 4 donors; two females, two males, age 29-67 years) from the lateral tibia head during high tibial closed wedge osteotomy as described previously [3]. The ethics committee of the Charité-Universitätsmedizin Berlin approved the study. In brief, spongious bone chips were cut into small fragments and digested for 4 h at 37°C using 256 U/mL collagenase XI (Sigma, St. Louis, MO, USA). The fragments were placed in Primaria™ culture flasks (Becton and Dickinson, Franklinlakes, NJ, USA) and cultured in DME-medium (Biochrom, Berlin, Germany) containing 10% human serum (German Red Cross, Berlin, Germany), 100 U/mL penicillin, 100 μg/mL streptomycin, 2 mM L-glutamine (all Biochrom), and 2 ng/ml fibroblast growth factor-2 (Tebu-bio, Boechout, Belgium). Cells that reached 80-90% confluence were subcultivated using trypsin in PBS (0.05% v/v, Biochrom) and re-plated at a density of 6,000 cells/cm^2^. Medium was exchanged every 2-3 days.

### Flow cytometric analysis

CSP (250,000 cells, passage 3) were washed in PBS/0.5% BSA and incubated with monoclonal mouse anti-human labeled antibodies CD34-Phycoerythrin (PE), CD73-PE, CD166-PE, CD45-Fluorescein-iso-thio-cyanate (FITC), CD90-FITC and CD105-FITC (all Becton and Dickinson) for 15 minutes. Staining of cell surface antigens was analyzed using FACS Calibur (Becton and Dickinson). Apoptotic cells were excluded from analysis using propidium iodide (PI). CD34 stained cells served as isotypic negative control.

### Collection of synovial fluid (SF)

Rheumatoid arthritis (RA) SF was obtained by joint puncture (n = 7; six female, one male; mean age 42 years; mean DAS 28 5.8; mean ESR (1 h) 58; mean CrP 5.8 mg/dL) from patients with an acute inflammatory phase diagnosed according to the revised American College of Rheumatology criteria for the Classification of RA [21]. Four donors with RA received disease modifying anti-rheumatic drugs (DMARD), three donors received steroidal anti-inflammatory drugs (SAID), three donors received non-steroidal anti-inflammatory drugs (NSAID), and two donors were treated with anti-tumor necrosis factor-α (anti-TNF therapy). None of the patients received intra-articular therapy. Osteoarthritis (OA) SF was obtained by joint puncture (n = 7; six females, one male; mean age 64 years; mean ESR (1 h) 12; mean CrP 0.7 mg/dL) from patients diagnosed according to the American College of Rheumatology criteria for the classification and reporting of OA [22]. Four of the donors with OA received NSAID and three donors had no medication at the time of joint puncture. None of the OA donors received intra-articular therapy. Normal (ND) SF was obtained post mortem within 24 hours from organ donors (n = 7; three females, four males; mean age 61 years). By visual inspection of the joint and in particular the articular cartilage, normal donors with joint diseases were excluded. All procedures were performed in consent with the ethics committee of the Charité-Universitätsmedizin Berlin.

### Assessment of mesenchymal lineage differentiation potential of CSP

For osteogenic and adipogenic differentiation, CSP (n = 4 donors) were plated with a density of 5,000 cells in 6-well culture plate (Becton and Dickinson). For osteogenic differentiation, confluent monolayer cultures were stimulated with low-glucose DME-medium containing 10% human serum and osteogenic supplements (0.1 μM dexamethasone, 50 μM L-ascorbic acid-2-phosphate, 10 mM β-glycerophosphate; (all Sigma) [23]. Cells were cultured for 18 days and medium was changed every other day. For adipogenic differentiation, cells were stimulated 3 days post confluence with high-glucose DME-medium containing 10% human serum and adipogenic supplements (1 μM dexamethasone, 200 μM indomethacin, 50 μM 3-isobutyl-1-methylxanthin; (all Sigma) [24]. Cells were cultured for 20 days. Controls were maintained without adipogenic or osteogenic supplements. Chondrogenic differentiation of CSP (passage 3) was performed under serum-free conditions in high-density pellet cultures (n = 4 donors, 250,000 cells/pellet) as described previously [25]. Chondrogenesis was induced by adding 10 ng/ml transforming growth factor beta 3 (TGFB3, R&D Systems, Minneapolis, MN, USA). The medium was exchanged every 2-3 days and cells were maintained for up to 28 days. Controls were maintained without TGFB3 supplements.

### Chondrogenic differentiation in the presence of human synovial fluid

To evaluate the influence of RA, OA and ND-SF on chondrogenic differentiation of CSP, pellets were treated with DME-medium (ITS + 1 (Insulin-Transferrin-Selenium), 0.1 μM dexamethasone, 1 mM sodium pyrovate, 0.17 mM ascorbic acid-2-phosphate, 0.35 mM proline (all Sigma) supplemented with 5% of the respective SF (pooled from seven donors, equal amounts) without TGFB3. Pellets cultured in DME-medium in the absence of SF served as controls. The medium was exchanged every 2 -3 days and cells were maintained for up to 28 days.

### Histochemical and immune-histochemical staining

From all donors (n = 3 cell pellets, n = 3 sections per cell pellet for n = 4 donor), cryosections (6 μm) were made and subsequently stained using a primary rabbit anti-human type II collagen antibody (Acris, Hiddenhausen, Germany). Therefore, sections were incubated for 40 minutes with antibody, colorimetrically detected by 3-amino-9-ethylcarbazole (EnVision™, Dako, Glostrup, Denmark) and counterstained with hematoxylin (Merk, Darmstadt, Germany). In addition, proteoglycans of CSP treated with SF (n = 3 cell pellets, n = 3 sections per cell pellet for each individual experiment) were visualized by staining Alcian Blue 8GS (Roth, Karlsruhe, Germany) at pH 2.5, followed by counterstaining with nuclear fast red (Sigma). Osteogenic cells were detected by staining of mineralized matrix components according to von Kossa. Intracellular lipid vacuoles in adipogenic cultures were visualized using Oil Red O staining (Sigma).

### Polymerase chain reaction (PCR)

As indicated, total RNA from 20 pellets per experiment was isolated as described previously [26]. Subsequently, total RNA (3 μg) was reversely transcribed with the iScript cDNA Synthesis Kit according to the manufacturer's instructions (BioRad, München, Germany). The relative expression level of housekeeping gene glyceraldehyde-3-phosphate dehydrogenase (GAPDH) was used to normalize samples. Real-time RT PCR using i-Cycler PCR System (BioRad) was performed with 1 μL of each cDNA sample in triplicate using the SYBR green PCR Core Kit (Applied Biosystems, Foster City, CA, USA). Relative quantification of marker gene (Table 1) expression was performed and is given as percentage of the GAPDH product or as fold change according to the ΔCT-method [27]. Differential expression was considered at fold change of FC > 2 or FC < 2.

**Table 1:** Oligonucleotides used for gene expression analysis

Gene name	Gene symbol	Oligonuclotides (5'→3') (Up/Down)	Product size (Base Pairs)
Glyceraldehyd-3-Phosphate dehydrogenase	GAPDH	GGC GAT GCT GGC GCT GAG TAC/TGG TCC ACA CCC ATG ACG A	149

Aggrecan	AGC1	CCA GTG CAC AGA GGG GTT TG/TCC GAG GGT GCC GTG AG	146

Cartilage oligomeric matrix protein	COMP	GGG TGG CCG CCT GGG GGT CTT/CTT GCC GCA GCT GAT GGG TCT C	116

Link-protein	LINK	GCG TCC GCT ACC CCA TCT CTA/GCG CTC TAA GGG CAC ATT CAG TT	145

Type IIα1 collagen	COL2A1	CCG GGC AGA GGG CAA TAG CAG GTT/CAA TGA TGG GGA GGC GTG AG	128

Type IXα1 collagen	COL9A1	AAT CAG GCT CTG AAG CTC ATA AAA/CCT GCC ACA CCC CCG CTC CTT CAT	100

Matrix metalloproteinase 1	MMP1	TAC ATG CGC ACA AAT CCC TTC TAC C/GAA AAA CCG GAC TTC ATC TCT GTC G	126

Matrix metalloproteinase 2	MMP2	TCC CTG CCC CTC CCT TCA AC/CCT TTC CAG CAG ACA CCA TCA CC	196

Matrix metalloproteinase 13	MMP13	CAA AAA CGC CAG ACA AAT GTG ACC/GAT GCA GGC GCC AGA AGA ATC T	105

Tissue inhibitor of metalloproteinases 1	TIMP1	GGC TTC TGG CAT CCT GTT GTT G/ACG CTG GTA TAA GGT GGT CTG GTT G	160

Tissue inhibitor of metalloproteinases 2	TIMP2	CTA GGG CAG ACT GGG AGG GGG AGG GTA TC/TGG AGG GGT CTG GTG GAG TTG TAT	143

## Results

### Morphology and cell surface antigen pattern of human CSP

**Figure 1:**
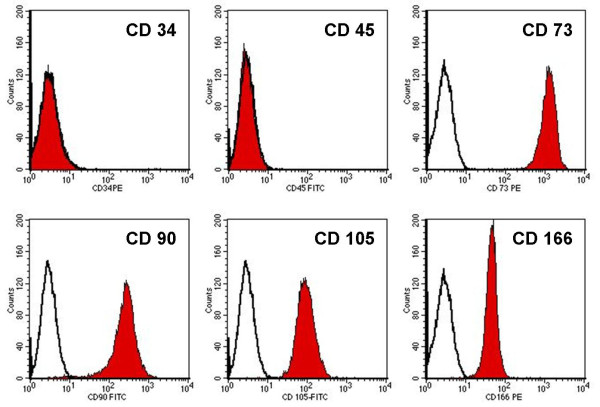
**FACS analysis of cultured CSP**. Histograms obtained from FACS analysis of culture expanded CSP. Cells were positive for the surface antigens CD 73, CD 90, CD 105 and CD 166 and negative for CD 34 and CD 45. The illustration is representative for all donors in FACS analysis.

Cultured primary CSP presented a spindle-shaped, non-granular fibroblast-like morphology and formed colonies. Subcultured cells grew in monolayer, maintained a stable fibroblast-like morphology with no signs of granulation. Expanded CSP showed typical cell surface antigens known from mesenchymal stem and progenitor cells. All CSP were homogenously positive (92%-100%) for CD75, CD90, CD105 and CD166. Cells were negative for the hematopoietic antigen CD34 (0.1% positive cells) and for the leukocyte common antigen CD45 (0.1% positive cells) (Figure 1).

### Differentiation potential of CSP

**Figure 2:**
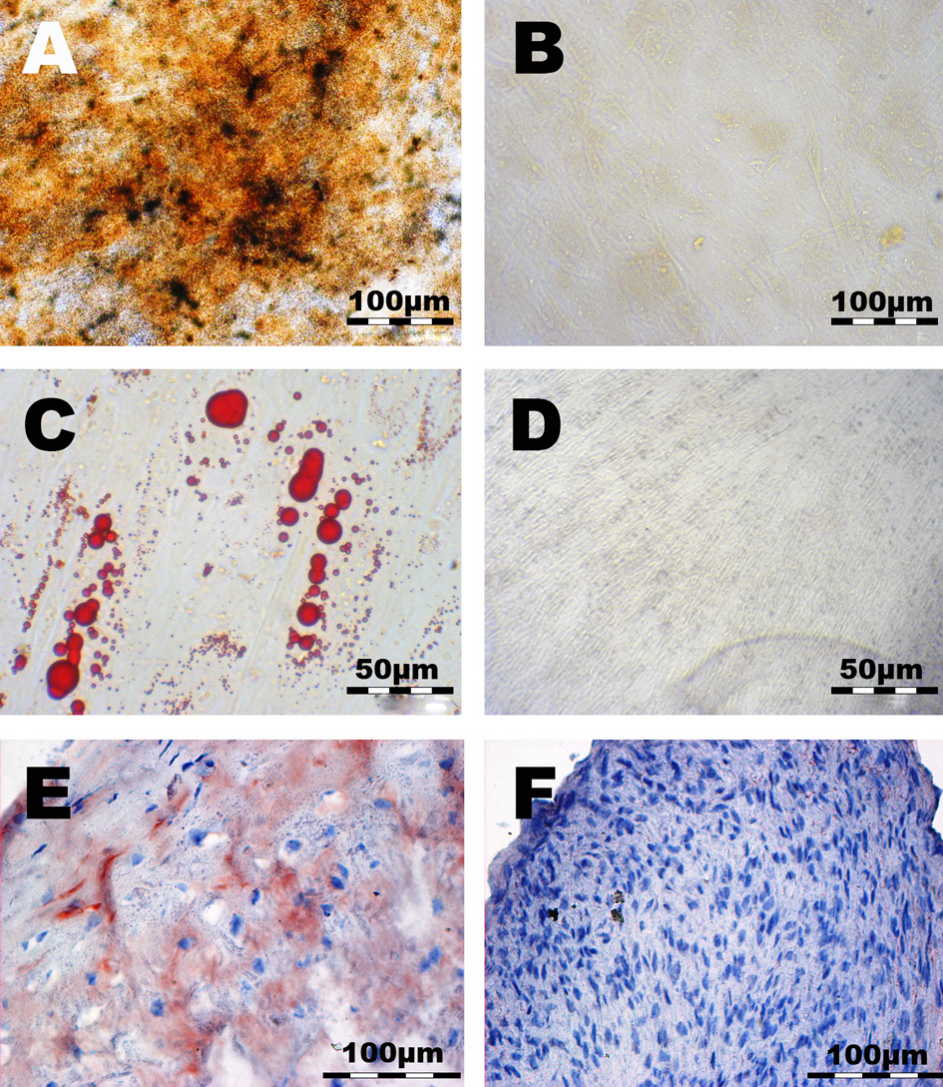
**Assessment of mesenchymal lineage differentiation potential of CSP**. Von Kossa staining for documentation of matrix mineralization showed **(A) **that, at day 18, mineralization was evident in osteogenic induced cultures. **(B) **In non-induced cultures no matrix mineralization was detected. Oil Red O staining for documentation of adipogenic differentiation showed **(C) **that, at day 20, lipid vacuoles were evident in adipogenic-induced cultures. **(D) **In non-induced cultures no lipid vacuoles were detected. Type II collagen staining showed **(E) **that, at day 28, type II collagen was evident in chondrogenic induced CSP. **(F) **In non-induced cells no type II collagen was detected. These illustrations are representative for all donors.

The potential of CSP to undergo osteogenic, adipogenic and chondrogenic differentiation was documented by visualization of matrix mineralization by von Kossa staining, by visualization of lipid vesicles by Oil Red O staining and by visualization of type II collagen by immune-histochemistry (Figure 2). At day 18, von Kossa staining showed in CSP-cultures stimulated with osteogenic supplements zones of high calcium content (A). In non-simulated controls no matrix mineralization was detected (B). At day 20, Oil Red O staining showed that lipid-filled vesicles were present in CSP stimulated with adipogenic supplements (C). In non-stimulated controls no lipid vesicles were detected (D). Immune-histochemical staining of CSP pellets stimulated with TGFB3 showed that CSP developed a dense tissue, rich in viable cells and deposition of cartilage-specific type II collagen, at day 28 (E). In absence of TGFB3, pellets showed no type II collagen deposition (F).

### Chondrogenic differentiation with synovial fluid

**Figure 3:**
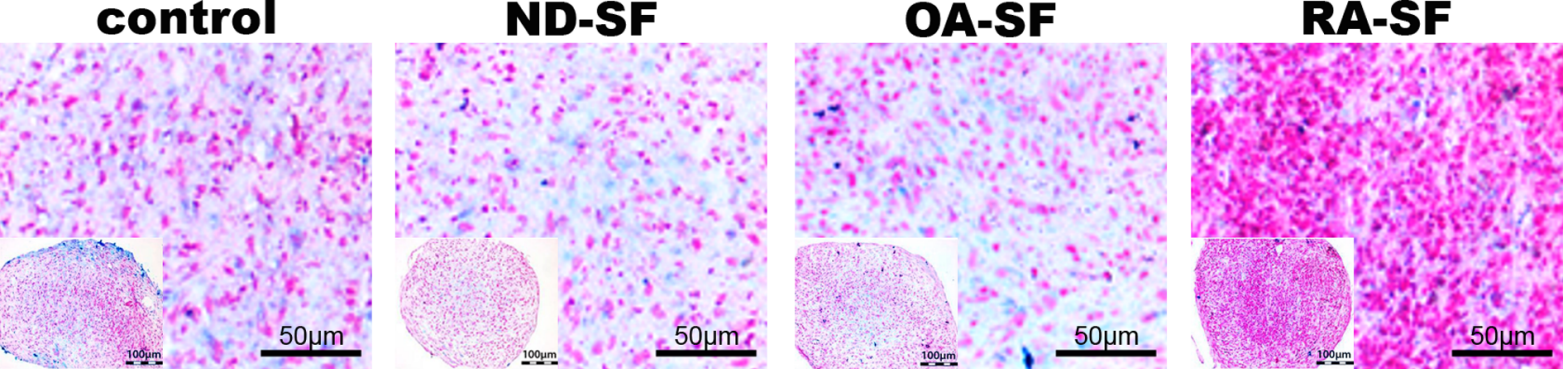
**Alcian blue staining of CSP pellet cultures**. Alcian blue staining of CSP pellet cultures cultured in the presence of synovial fluid from ND, OA or RA donors, at day 28.

**Figure 4:**
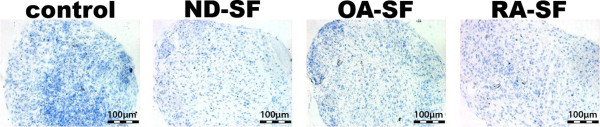
**Type II collagen staining of CSP pellet cultures**. Type II collagen staining of CSP pellet cultures at day 28 cultured without TGFB3 but in presence of synovial fluid. The illustration is representative for all other samples, which were taken at day 1, 7, 14 and 21.

Histochemical staining of CSP pellets showed that control pellets and pellets cultured in presence of ND or OA-SF (without TGFB3) evolved a dense tissue, rich in viable cells and marginal proteoglycan in the centre area (Figure 3). However, pellets cultured in presence of RA-SF evolved a more compact tissue compared to pellets cultured in the presence of ND or OA-SF. No proteoglycan was detectable in pellets treated with RA-SF. Immune-histological staining of type II collagen showed that control pellets and pellets cultured in presence of SF (ND, OA or RA) was void of type II collagen (Figure 4) at day 28. There was also no type II collagen detectable at day 1, 7, 14, 21 (data not shown).

### Gene expression analysis of human subchondral CSP undergoing chondrogenic differentiation

**Figure 5:**
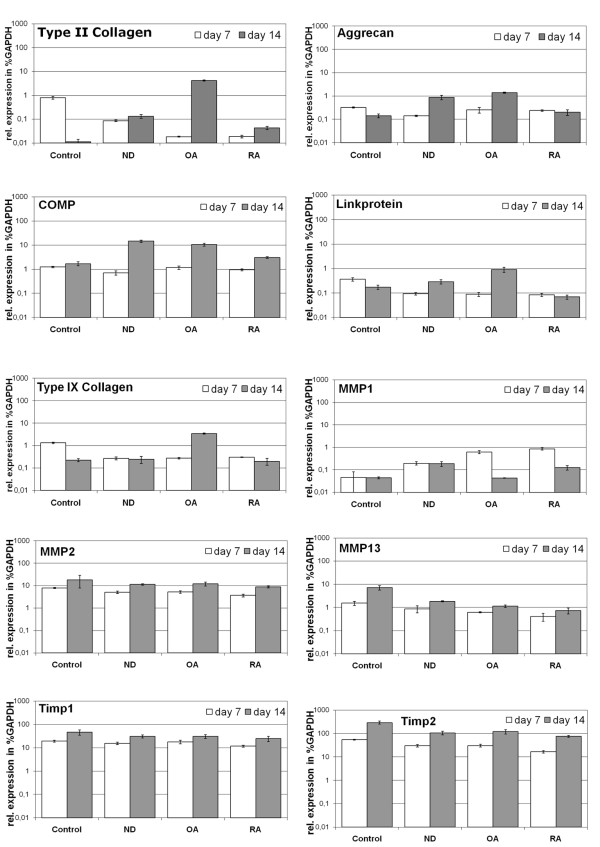
**Gene expression analysis of CSP pellet cultures**. Semi-quantitative real-time gene expression analysis of human CSP pellets treated with synovial fluid from ND, OA and RA donors. Chondrogenic differentiation of CSP was analyzed by gene expression analysis (n = 3) of the typical chondrocytic marker genes aggrecan, cartilage oligomeric matrix protein (COMP), link-protein type II and type IX collagen. Matrix remodelling was assessed by gene expression analysis of the matrix metalloproteinases (MMP)-1, -2 and -13 as well as their inhibitors (TIMP)-1 and -2. The expression level of marker genes was calculated as percentage of the expression level of the housekeeping gene GAPDH. The mean of each triplicate is plotted and the error bars represent SD.

Gene expression analysis of CSP pellets stimulated with synovial fluid without TGFB3 showed differences in the expression of type II collagen, proteoglycans, and link-protein, which was detected at day 14 of chondrogenic differentiation (Figure 5). CSP stimulated with ND or OA-SF showed induction of the gene expression of the chondrogenic marker gene type II collagen (from 0.1% to 0.4%-4%) compared to the control. CSP stimulated with ND or OA synovial fluid showed elevated expression of the chondrogenic marker genes aggrecan (from 0.15% of the expression level of GAPDH in controls to 1-1.5% in CSP treated with ND-SF or OA-SF), the cartilage oligomeric matrix protein (COMP) (from 0.3% to 1.4-1.6%) and the link-protein (from 0.15% to 0.3-0.9%). RA-SF similar induced the expression of aggrecan, COMP and link-protein of CSP compared to CSP treated without TGFB3 and synovial fluid (control). Additional OA-SF induced the expression of type IX collagen (from 0.2% to 3.4%). Whereas, the gene expression of type IX collagen in ND or RA-SF stimulated cells remained on the same level as the control.

Compared to control, CSP stimulated with ND-SF or RA-SF showed an increase in the expression of genes related to matrix remodeling like MMP1 (from 0.05% to 0.1-0.2%), whereas CSP induced with OA-SF presented a similar expression of the MMP1 gene compared to the control. Furthermore CSP stimulated with SF's presented a similar expression of the MMP2 and TIMP1 genes compared to the control. Compared to control, the expression of MMP13 (from 7.2% to 0.8-3%) and metalloprotease inhibitor TIMP2 (from 293% to 96-120%) was repressed in CSP co-stimulated with synovial fluids only.

**Figure 6:**
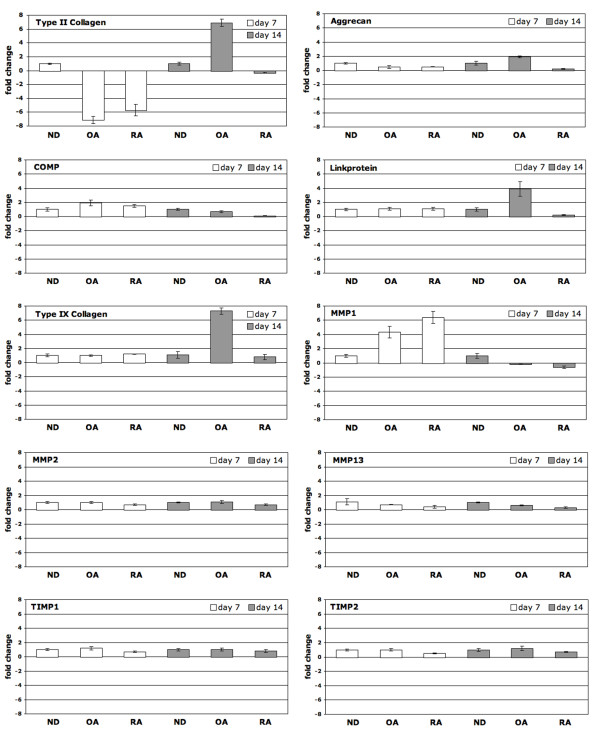
**Effect of rheumatoid arthritis and osteoarthritis synovial fluid on the chondrogenic differentiation of CSP**. Differential expression of maker genes in CSP treated with synovial fluid from ND, OA and RA donors. To assess the effect of rheumatoid arthritis and osteoarthritis on the "normal" chondrogenic differentiation of subchondral progenitors, the expression profile of the typical chondrogenic marker genes aggrecan, cartilage oligomeric matrix protein (COMP), link-protein type II and type IX collagen was evaluated. Matrix remodelling was assessed by gene expression analysis of the matrix metalloproteinases (MMP)-1, -2 and -13 as well as their inhibitors (TIMP)-1 and -2 and is given as fold change compared to the expression level in CSP treated with normal synovial fluid. The mean of each triplicate well is plotted and the error bars represent SD.

Since the presence of ND-SF represents the physiological condition in healthy joints, the gene expression levels of the respective markers in the diseased condition were calculated in relation to the 'normal situation' (Figure 6). At day 7, compared to CSP treated with ND-SF, SF from OA induced COMP (FC = 2.0) and MMP1 (FC = 4.3), whereas the marker gene type II collagen (FC = -7.1) was repressed. SF from RA induced MMP1 (FC = 6.4), and the marker gene type II collagen (FC = -5.7 was repressed. At day 14 compared to CSP treated with ND-SF, SF from OA induced the marker genes type II collagen (FC = 6.9), aggrecan (FC = 2.0), link-protein (FC = 3.9) and type IX collagen (FC = 7.3). SF from RA showed no significant changes in chondrogenic marker gene levels compared to ND-SF at day 14. Nevertheless, a tendency of increase of type II collagen, aggrecan, COMP and link-protein can be observed at day 14 compared to ND-SF.

## Discussion

This study was performed to clarify some preliminary effects on the *in vitro *chondrogenic differentiation potential of cortico-spongious progenitor cells (CSP) affected by synovial fluid (SF) obtained from healthy donors (ND) and patients with OA or RA. For this purpose, CSP were analyzed histochemically for aggrecan and type II collagen. Also gene expression analyses of cartilage specific marker genes were performed. Because there is a lag of basic understanding in the principles in cell biology and especially in the interaction of the cells with the surrounding environment that controls and directs function. Few studies indicate that synovial fluid (SF) compounds affect the *in vivo *environment of cartilage in osteoarthritis (OA) and rheumatoid arthritis (RA) patients [16,28]. It could be demonstrated that RA-SF impaired the chondrogenic differentiation of human subchondral progenitor cells [4]. Therefore, it remains unclear, if engineered tissue or manufactured material for cartilage repair can be placed in patients with OA or RA. It is known that under normal conditions the cartilage matrix is subjected to a dynamic remodeling process in which low levels of degradative and synthesizes enzyme activities are balanced, such that natural turnover of cartilage is maintained [29]. In OA and RA cartilage, however, matrix degrading enzymes are over expressed, shifting this balance to increased degradation resulting in loss of collagen and proteoglycans from the matrix [17,30].

To verify the homogeneity of cell population, the functional characteristics of the isolated CSP from each donor were analyzed. The analysis contains the investigation of certain surface antigens and the multi-differentiation potential. The results showed that all human CSP isolated from tibia grown exponential in the presence of human serum and present the typical mesenchymal progenitor cell related cell surface antigen pattern. Also, CSP have the ability to undergo osteogenic, adipogenic and chondrogenic differentiation. These findings confirm with the international society for cellular therapy position statement, which define the minimal criteria for mesenchymal progenitor cells [31] and some published articles which deal with the characterization of mesenchymal progenitor cells derived from subchondral bone [3,5,6].

**Figure 7:**
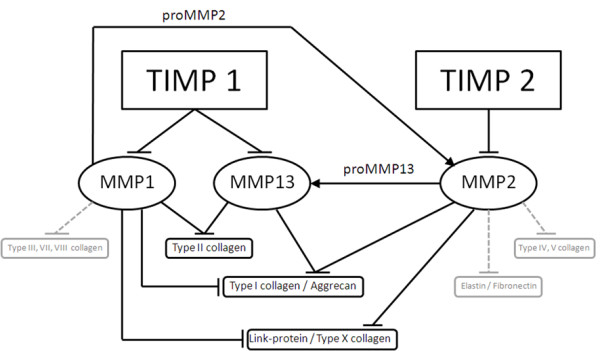
**Pathway chart of the selected MMP/TIMP interaction**. Overview on distinct MMPs with their proteolytic substrates and outline of interaction of TIMP inhibition and MMPs.

It is believed that cytokines and growth factors play an important role in the pathophysiology of OA and RA [13,28]. They are closely associated with functional alterations in synovium, cartilage and subchondral bone and are produced both spontaneously and following stimulation by joint tissue cells. Moreover, several of these agents (e.g. α2-macroglobulin (α2 M), metalloproteinases (MMP) and proteinase inhibitors TIMP) have been identified in human synovial fluid [17,32]. The MMP are the major proteolytic enzymes that facilitate tissue remodeling in both physiological and pathological situations. The MMP do indeed have the combined ability to degrade the major components of the cartilage extracellular matrix such like type II collagen and aggrecan (Figure 7) [29,33]. However, it should be taken into account that the alterations found in OA and RA compared to the healthy situation may not be represented by the synovial fluids used in the current study. A clear limitation might be that the SF from normal donors was obtained post-mortem and therefore may show non-physiological changes in its composition compared to a healthy or traumatic SF. In addition, disease-related medical treatment of the donors may lead to alterations in the SF composition. Therefore, further studies have to elucidate whether SAID or NSAID have an impact on the composition of SF and in turn of the chondrogenic differentiation of progenitors in the presence of the respective SF.

The gene expression analysis of chondrogenic marker genes in CSP treated with synovial fluid showed that the cartilage marker genes COMP, link-protein, aggrecan and type II collagen are slightly increased after 14 days in high-density cultures of CSP under serum-free conditions. These findings underline the fact that synovial fluid contains factors such like hyaluronic acid which promotes and enhances the development of cartilage by mesenchymal stem and progenitor cells [7]. Synovial fluid also contains factors of the TGFB sub family, fibroblast growth factors (FGF) and bone morphogenetic proteins (BMP), which are able to induce the chondrogenic differentiation [28,32]. However, the stimulation with synovial fluid was not sufficient to detect some aggrecan or type II collagen on protein level, but it was detectable on mRNA level. To clarify the question of dependency of the MMP/TIMP production, the gene expression data of CSP cultured with synovial fluid (ND, OA or RA) showed that the mRNA level is decreased for MMP1 and MMP3 during cultivation of CSP treated with OA-SF and RA-SF compared to CSP treated with ND-SF. However, the mRNA level of MMP2, TIMP1 and TIMP2 showed a similar expression during the cultivation of CSP treated with ND-SF, OA-SF or RA-SF. Therefore, the CSP are not responsible for the MMP/TIMP imbalance in RA and OA synovial fluid. As a consequence it can be assumed that only synovial cells are involved in the MMP/TIMP ratio balance or imbalance. This findings correlate with a published article, which supposed that chondrocytes are not involved in the development of the MMP/TIMP ratio in SF [34].

Moreover, this study showed that supplementation of the chondrogenic culture medium (without TGFB3) with synovial fluid from OA induced the marker genes aggrecan, type II collagen, link-protein and type IX collagen at day 14, compared to CSP treated with ND-SF. So it might be possible that the same mechanism were activated, which is responsible in OA cell cluster formation for the expression and synthesis of new components of extracellular matrix [34,35]. This suggests that wound healing, including collagen synthesis, occurs in damaged OA cartilage. Additionally, the results demonstrated that ND-SF also induces chondrogenesis on RNA level of CSP. In contrast, the supplementation of the chondrogenic culture medium with RA-SF reduced chondrogenesis of CSP compared to ND-SF. Reflecting results obtained in this study and the knowledge from the literature, a RA environment reduces the cartilage regeneration [4]. So it might be possible to use tissue engineering strategies for cartilage repair in the early stage of osteoarthritis. But the etiology of the pathogenic cartilage defect needs to be eliminated before [36]. Also the beginning of an inflammatory process should be treated with anti-inflammatory therapeutics. Extremely questionable is the use of cell based strategies for cartilage replacement in RA patients. In this study it was demonstrated that RA-SF has an effect on the mRNA level of chondrogenesis. To further clarify these facts an *in vivo *OA and RA model is necessary.

## Conclusions

In conclusion our study showed that rheumatoid arthritis synovial fluid impairs the chondrogenic differentiation of human subchondral progenitor cells, whereas osteoarthritis fluid induced after 14 days the mRNA level of aggrecan, type II collagen, link-protein and type IX collagen compared to rheumatoid synovial fluid. These results indicate that an inflammatory environment found in rheumatoid arthritis negatively affects the chondrogenesis during cartilage repair, while an osteoarthritis environment may not impair, but delay the cartilage repair by subchondral progenitor cells.

## Competing interests

Krüger, J.P.; Endres, M.; Neumann, K. and Kaps, C. are employees of TransTissue Technologies GmbH (TTT) which develop regeneration therapies based on mesenchymal cell products.

## Authors' contributions

JPK: Analysis and interpretation, data collection and writing the article. ME: Assessment of lineage differentiation and data collection. KN: Flow cytometric analysis (FACS) and data collection. BS: Collection and classification of the synovial fluid. LM: Collection and preparation of the precursor material for CSP cell isolation. TH: Collection and classification of the synovial fluid. CK: Design of the study and critical revision of the article.
